# Thrombotic Microangiopathies

**DOI:** 10.5402/2012/310596

**Published:** 2012-07-25

**Authors:** Mohamed Radhi, Shannon L. Carpenter

**Affiliations:** Pediatric Hematology/Oncology/Stem Cell Transplant, Children's Mercy Hospital, Kansas City, MO 64108, USA

## Abstract

Thrombotic microangiopathy results from thrombotic occlusion of the microvasculature leading to fragmentation of red blood cells, profound thrombocytopenia, and a microangiopathic hemolytic anemia with elevation of lactate dehydrogenase and negative direct Coomb's test. This constellation of clinical and laboratory findings is not due to one disease entity; rather, it represents a variety of underlying diagnoses. Among the major disease entities are TTP/HUS, which can be congenital or acquired, bacterial infections, medications, vascular or endothelial pathology like Kasabach-Merritt phenomenon, and stem cell transplantation. In this paper, we offer a review of some of the major causes of thrombotic microangiopathy.

## 1. Introduction

Thrombotic microangiopathy (TMA) results from red cell fragmentation and platelet trapping with subsequent microangiopathic hemolytic anemia and thrombocytopenia and represents a final common pathway of a multitude of clinical syndromes. In this paper, we attempt to summarize the salient features of many of these clinical entities and provide suggestions to help guide the efforts to differentiate between them. This is by no means meant to be an exhaustive discussion of these disorders, but rather strives to be a useful summary of some of the important causes of TMA.

## 2. Kasabach-Merritt Phenomenon

Kasabach-Merritt phenomenon (KMP) typically describes thrombocytopenia and consumptive coagulopathy in the presence of a vascular tumor. KMP is very rare, occurring in only 0.3% of congenital hemangiomata. It was initially described as being associated with a rapidly enlarging infantile hemangioma [1]. However, further research has shown that KMP is most commonly associated with the rare benign vascular tumors Kaposiform Hemangioendothelioma (KHE) and Tufted Angioma (TA) [2, 3]. More than 50% of the cases of KHE and TA occur in the first year of life. 

KHE and TA tend to occur on the extremities, neck or trunk, but can sometimes involve the retroperitoneum. For this reason, thrombocytopenia in a newborn unexplained by other routine testing (such as for sepsis) should prompt imaging for occult vascular lesions. The pathogenesis of this condition has not been fully elucidated, but to the best of our understanding, the abnormal endothelium of these tumors results in platelet trapping, fibrin deposition with subsequent red blood cell damage, and a consumptive coagulopathy. Thrombocytopenia is usually severe and hypofibrinogenemia is present. While microangiopathic features are often apparent, anemia is not a common presenting sign unless significant bleeding has occurred. Bleeding into the lesion leads to rapid enlargement of the tumor, continuing the cycle of platelet trapping and activation with consumption of clotting factors [1]. KMP has been reported as having 30–40% mortality, usually due to uncontrolled bleeding, but also secondary to cardiac failure or invasion of vital structures by the lesion [4]. 

Historically, interferon alpha has been used successfully as treatment for KMP, but the side effect of irreversible spastic diplegia, particularly in children under 1 year of age, has led to this drug falling out of favor, though it is still used in some cases. First-line treatment now typically relies upon corticosteroids and vincristine, though no prospective randomized trials have been completed comparing this regimen to an alternate treatment [5, 6]. Use of other chemotherapies such as cyclophosphamide, actinomycin-D, and methotrexate and of antiplatelet treatments such as ticlodipine has been reported [7, 8]. There are studies currently underway investigating the benefit of novel therapies such as rapamycin. Surgical resection, radiation, and embolization of the lesions have also been reported [4, 9, 10].

## 3. Thrombotic Thrombocytopenic Purpura

Thrombotic thrombocytopenic purpura (TTP) has classically been defined as the pentad of microangiopathic hemolytic anemia, thrombocytopenia, fever, and neurological and renal compromise. However, the diagnosis requires only microangiopathic anemia and thrombocytopenia, with or without ischemic organ damage, and not attributable to an alternate recognizable cause. This has of course increased both the prevalence and diversity of the disease. The estimated incidence of TTP is 2–10 per million/year [11]. 

Untreated, the mortality of TTP was historically as high as 80–90%. With the use of plasma exchange treatment, the overall mortality has been reduced to 20–30% [12]. The term TTP (or secondary TTP) has also been used to refer to microangiopathic hemolytic anemia associated with vasculitis, infection, pregnancy, medication, or which develops post-stem-cell transplant [13]. We will discuss those entities separately. For the purposes of this section of this paper, we will focus on congenital and idiopathic acquired TTP. 

Recent research has illuminated some of the underlying pathophysiology associated with this disorder, though many questions still remain. TTP is the result of thrombi in the microcirculation, leading to thrombocytopenia and microangiopathic hemolytic anemia due to red cell damage within the microthrombi [14]. One of the most exciting developments in the field of TTP has been the identification of the von Willebrand factor-cleaving protease, ADAMTS13 (A Disintegrin and Metalloprotease with ThromboSpondin type 1 repeats). This enzyme is responsible for the cleavage of extra large multimers of von Willebrand factor (VWF) into smaller moieties. The lack of this enzyme, either constitutionally or acquired, has been linked to the development of TTP [15, 16]. This phenomenon does not account for all of the cases of TTP, however, and further research is required to explain the complete link between this enzyme and the clinical variability of the disease [17]. 

Idiopathic TTP typically occurs due to the development of autoantibodies to ADAMTS13, usually type G immunoglobulins [18]. Measurement of ADAMTS13 activity and inhibitor titers has improved the diagnosis of TTP. Approximately 20–50% of patients with idiopathic TTP will experience a relapse [12]. Patients' risk of relapse is somewhat predicted by very low ADAMTS13 levels at diagnosis. However, variations in ADAMTS13 activity and inhibitor levels after treatment has been completed have not been consistently predictive of pending relapse, putting the utility of following these levels into question [12, 19, 20]. 

Plasma infusion or exchange is the mainstay of treatment for acquired TTP [12, 21]. Additional treatment modalities such as steroids, vincristine, cyclophosphamide, antiplatelet agents, and Rituximab (anti-CD20 antibody) have also been used to varying success [22]. 

Inherited TTP, also known as Upshaw-Schulman Syndrome, results from genetic mutations in the ADAMTS13 gene and represents approximately 10% of those patients with TTP. This is an extremely rare disorder, estimated to have an incidence of only 1 : 1,000,000 [17]. It is inherited in an autosomal recessive pattern, sometimes homozygously, but more often due to compound heterozygosity, and penetrance can be variable within the same family. Most patients present in infancy, but there are patients who have presented during the second or third decades of life, and there are individuals who are known to carry mutations who show no symptoms. This has led to the hypothesis that, in some individuals carrying 2 mutations, an additional triggering event in conjunction with low ADAMTS13 levels is required to develop TTP [11, 14].

Transfusions of normal plasma are the standard of treatment for congenital TTP. Recombinant and plasma-derived ADAMTS13 replacement products are in development [23]. In addition, a novel aptamer which blocks platelet activation in TTP is currently under investigation and has completed phase I studies [24, 25]. Gene therapy has also been suggested as a treatment for this disorder, though research is still in the preclinical stages [26]. 

## 4. Hemolytic Uremic Syndrome

Hemolytic uremic syndrome (HUS) is characterized by microangiopathic hemolytic anemia and thrombocytopenia with accompanying renal impairment [27]. It can be divided into 3 subtypes: diarrhea-associated HUS (D+HUS), pneumococcal (also known as atypical or diarrhea negative) HUS, and congenital HUS (which has also been referred to as atypical). While the three entities share clinical features, the underlying epidemiology, pathogenesis, and treatment are markedly different. Like TTP, the term HUS has been used to refer to a spectrum of thrombotic microangiopathy caused by medications or associated with pregnancy or other clinical conditions [13, 28]. We will only address the three entities listed above in this section of the paper.

Diarrhea-associated HUS (D+HUS) is the most common subtype of HUS. It usually occurs in children and is most commonly caused by infection with Shiga-toxin producing *Escherichia coli *O157 (STEC) [29]. The mean incidence of HUS in children under 18 years of age ranges from 0.28 to 0.71 cases per 100,000 persons. The disease is more common in those <5 years of age, in whom the mean incidence has been reported between 0.85 and 1.87 cases per 100,000 children [29, 30]. D+HUS is also the most common cause of acute renal failure in children in the USA. Mortality ranges from 1 to 33% with the highest mortality in those >60 years of age [29]. 

The pathogenesis of D+HUS results from the production of Shiga-toxins (Stx) by the infective *E. coli. * While the most common type of *E. coli *associated with this illness is the O157 : H7 strain, it is well known that other strains of *E. coli* may also produce Stx. STEC lead to a food- or water-borne diarrheal illness that can be bloody or nonbloody. The disease progresses to HUS due to the release of Stx in the intestine, which then are translocated across the gut epithelium into the circulation. The primary target of Stx is a unique digalactose moiety in the glycolipid Gb3. The primary locations of Gb3 in the human body are vascular endothelial cells, particularly in the glomeruli. Exposure to Stx damages the endothelium, resulting in the release of tissue factor and exposure of the platelets to extra large von Willebrand factor multimers and collagen. This then triggers activation of the platelets and the coagulation cascade and leads to thrombus formation. The impact of the actions of Stx in these locations results in microangiopathic hemolytic anemia and thrombocytopenia. In addition, Stx has also been shown to bind to and directly activate platelets via the Gb3 receptor [31]. HUS does not typically result in hypofibrinogenemia [32, 33].

Treatment for D+HUS relies mainly upon supportive care [21]. There have been recent studies regarding SYNSORB Pk, an agent composed of silicone dioxide particles linked to the Pk trisaccharide that is responsible for Stx binding to the endothelial cell surface. This is meant to block the translocation of Stx into the circulation. Monoclonal antibodies to Stx are also under investigation [32]. In experimental studies in mice these antibodies have been shown to neutralize Stx [33]. Studies in healthy volunteers have shown that they are well tolerated [34].

HUS associated with pneumococcal infection (D-HUS or P-HUS) is one of the atypical forms of HUS. It is much less common that D+HUS accounting for only approximately 5–10% of HUS cases overall, though it may be responsible for as many as half of D-HUS cases. It has been associated with a higher morbidity and mortality than D+HUS, though increased awareness and improved treatment has improved this finding. P-HUS tends to occur in very young children, with the average age typically under 1 year. There is a slight male preponderance. Most cases have fairly severe pneumococcal pneumonia and evidence of pleural effusion. Meningitis has also been reported. The overall mortality rate is currently approximately 10%, which is much improved from previous reports of mortality from 29–50% [35, 36].

*Streptococcus pneumoniae* release neuraminidase, an enzyme that results in desialylation of red blood cell membrane proteins and exposure of the Thomsen-Friedenreich cryptantigen (T-antigen). The T-antigen is a ubiquitous antigen on human blood cells to which all people have IgM antibodies. Exposure of this antigen leads to hemolysis, thrombocytopenia, and microthrombi. It is not clear if these symptoms are antibody mediated or are a result of another mechanism [35]. 

Treatment for pneumococcal-associated HUS relies on treatment of the underlying infection. In addition, supportive care with dialysis may be required for those patients in kidney failure. Plasma exchange therapy has also been used in a subset of patients. Many patients require blood product transfusion, though exposure to plasma should be limited because of the possibility of worsening the hemolysis due to infusion of IgM anti-T antibody [35]. Vaccination against pneumococcus would reasonably be expected to reduce the prevalence of P-HUS, since the most common serotypes associated with HUS are included in the vaccine. However, it has recently been shown that serotype 19A may have emerged as an important invasive pathogen leading to P-HUS. This serotype is not included in the PCV7 [35].

Another important type of atypical HUS (aHUS) is associated with complement deregulation. This may present congenitally or later in life. It is thought that up to one-third to one-half of patients with aHUS have a mutation in one of the complement regulatory proteins. The deficiency of factor H, a regulatory glycoprotein in the alternative complement pathway, leads to a form of congenital HUS. Factor H provides protection from complement activation. The absence leads to overactivity of the alternative complement pathway and subsequent features of HUS. Mutations causing deficiencies in factor I, C3, and membrane cofactor protein CD46 have also been reported [31, 37, 38]. Gain-of-function mutations in complement factor B and C3, have been identified in families with aHUS and persistent activation of the alternative complement pathway. Families carrying mutations show incomplete penetrance of disease and may be modulated by the presence or absence of other complement gene variants [38–40]. Autoantibodies to factor H which develop in individuals with mutations in factor H related proteins 1 and 3 have also been implicated in the pathogenesis of the disease [41]. 

The prognosis for aHUS is grim, with mortality approaching 25% in the acute phase and as many as 50% of patients developing end stage renal disease. If transplantation is attempted, 60–100% of patients will experience a relapse, which almost inevitably results in loss of the renal graft [37]. Outcome is somewhat dependent upon the specific mutation leading to disease [36]. 

Treatment relies on infusion of plasma which contains the missing factor. In the case of those patients with autoantibodies, plasma exchange and immunosuppressive therapy have also been successful [41]. Recent studies regarding the use of the recombinant complement inhibitor eculizumab have been promising, particularly in those patients who have failed plasma-replacement therapy [37]. 

## 5. Transplant-Associated MAHA

Microangiopathic hemolytic anemia (MAHA), also called posttransplant thrombotic microangiopathy (TA-TMA) has been described as a complication of bone marrow transplantation, both autologous and allogeneic with variable incidence rates ranging from 0 to 74% depending on the diagnostic criteria used to make the diagnosis [42, 43]. It is also a well-known complication following solid organ transplants, such as kidney transplants, with a variable time from transplant to diagnosis and could be due to viral infections, arise de novo, or be related to the use of calcineurin inhibitors, mainly the microemulsion form of cyclosporine [37, 44]. TMA was observed in 26 of 188 patients with renal transplants with an incidence of 14% with the majority of these patients having no systemic manifestation of TMA. It has also been reported following liver transplantation [45]; five of 100 patients who received Living Donor Liver Transplant (LDLT) developed TMA with an incidence of 5% and at a median interval of 18 days following transplant. Another major factor in the development of TMA is drugs used for Graft Versus Host Disease (GVHD), mainly FK506 and cyclosporine [44] or with other GVHD prophylaxis [46]. Factors that can contribute to this condition include high-dose chemotherapy for autologous stem cell transplant (ASCT), conditioning regimen for allogeneic stem cell transplant, total body irradiation, and use of immune suppressive therapy [38, 47, 48]. 

The role of GVHD and renal involvement in the pathogenesis of TA-TMA has also been investigated [41]. Changsirikulchai et al. [49] reviewed 314 renal autopsies on patients who died after their first hematopoietic stem cell transplant over a 7-year period. Twenty percent of autopsies reviewed showed TMA, with an additional 15% with evidence of thrombus formation. The risk of TMA in that patient population increased 4-fold with incidence of acute GVHD, independent of the use of cyclosporine. The presence of acute GVHD was an independent risk factor in the development of TA-TMA in the multivariate analysis in addition to sex mismatch, higher doses of total body irradiation (TBI), and viral infections. 

In addition to the significant renal involvement with acute GVHD, TA-TMA has also been found to involve the gut tissue [50]. In a study of 16 patients with TA-TMA, all were found to have acute GVHD. Seven patients were retrospectively diagnosed with TA-TMA, while 9 were specifically investigated for it. All patients that were retrospectively diagnosed died as a result of increasing the immune suppression intensity due to the impression that it was purely gut GVHD, while 8 of the other nine patients improved following reduction of immune suppression. Six of those patients were alive one year following transplant. 

Infections have also been shown to be associated with MAHA after transplant; bacterial infection with *Helicobacter pylori* was found to be associated with six patients among 74 consecutive transplants through involvement of elevated cytokines [51]. Viral infections such as parvoB-19, CMV, and HHV-6 have also been implicated as a cause of TA-TMA. Typically postinfectious cases are due to reactivation of these viruses within the first 3–6 months after transplant. Viral infections should be considered in recipients of transplant who develop refractory cases of TA-TMA [39, 52, 53].

## 6. Pregnancy-Associated MAHA

Pregnancy-associated thrombotic microangiopathy is a rare but serious disorder that is associated with significant maternal and perinatal morbidity and mortality, due to the deposition of fibrin and platelet thrombi in the microcirculation of the placenta. It has been reported to occur in 1 : 25,000–1 : 100,000 pregnancies [54, 55]. Pregnancy can precipitate the disease for the first time or can exacerbate the recurrence of an existent condition [56, 57]. Pregnancy-associated microangiopathy typically occur during late pregnancy [58]; however, it has also been reported in the first trimester of pregnancy [59], in which case the patient was managed very intensely with continuous plasma therapy throughout the pregnancy. Both mother and fetus survived without complications. In reviewing the Oklahoma TTP-HUS registry in 335 patients, Terrell et al. [60] did not find any gender or race predilection that was associated with pregnancy-associated microangiopathy, as well as with hematopoietic stem cell transplant or drugs.

Martin et al. recently published data on the largest collection of pregnancy-associated microangiopathy [53] encompassing over 4 decades. One hundred and sixty-six patients with pregnancy-associated thrombotic thrombocytopenic purpra were reported on. Only patients who met specific diagnostic criteria were included in the study. Patients either had their first episode (*n* = 106) or recurrent episode (*n* = 32). Median time presentation was 23.7 ± 9 weeks and 22 ± 10 weeks, respectively. The only significant laboratory differences between both groups were hemoglobin and hematocrits (6.8 versus 11.4 g/dl and 19% versus 25%, resp.). Perinatal and maternal mortalities were high in both groups (32% versus 44% and 26% versus 10.7%); however, both mortality rates were significantly lower in the years after 1980 compared to before that year. One of the confusing clinical pictures was the association of pregnancy-associated TTP and HELLP syndrome (*H*emolysis, *E*levated *L*iver enzymes, *L*ow *P*latelets), which had both preeclampsia/eclampsia and TTP concurrently. This was reported in 29 of the 166 patients in the series and significantly increased maternal mortality from 21.8% to 44.4%. 

Pregnancy-associated microangiopathy can be confused with other medical and obstetrical conditions such as severe preeclampsia/eclampsia, HELLP syndrome, sepsis, and pregnancy-induced fatty liver changes [57, 61]. The confusion in making the diagnosis usually happens due to the non-specific clinical and laboratory criteria needed to make the diagnosis. This is more of a problem when the initial management is done by physicians who are not accustomed to taking care of these patients. Stella et al. reported on 14 cases of pregnancy-associated microangiopathy in 12 pregnancies by reviewing the charts of these patients [62]. 4/12 cases were evaluated in the ER initially and were given the diagnoses of domestic violence, gastroenteritis, ITP, and panic attack. 2/4 patients were discharged home multiple times before hospitalization. 8/12 patients were seen by obstetric services; however, all were given the wrong diagnosis, mainly HELLP syndrome, despite of being normotensive and having no proteinuria. Half of the patients in the series were diagnosed before 24 week gestation but only 3 had term live births.

 Over the last 2 decades, there has been increasing evidence that ADAMTS13 is significantly involved in the pathogenesis of pregnancy-associated microangiopathy. This could be due to congenital deficiency of the enzyme activity which is usually severe or due to the presence of inhibitors with less sever deficiency [63, 64]. However, some patients with pregnancy-associated microangiopathy were found to have a normal enzyme activity while other patients with TTP due to other clinical syndromes had low enzyme activity [64]. The congenital deficiency of the ADAMTS13 activity is also known as Upshaw-Schulman Syndrome (USS) [58]. In their research paper, Fujimura and colleagues [64] reported a series of 15 pregnancies with this syndrome. Thirty-seven patients were identified with the syndrome in 32 families. Nine females with a total of 15 pregnancies developed significant thrombocytopenia during the second half of their pregnancies leading to stillbirth in 8 out of total 16 babies. Eight of the nine women had heterozygous mutations and only one had a homozygous abnormality. 

Although ADAMTS13 enzyme deficiency (both congenital and acquired) is the main underlying mechanism in pregnancy-induced microangiopathy, dysregulation of alternative C3 convertase enzyme, due to mutations in the gene coding for the protein has also been shown to cause an atypical form of HUS during pregnancy (mainly with renal involvement and not due to Shiga toxin) [54]. In a retrospective analysis of 100 women with this rare and ill-defined type of HUS, it was found to be associated with pregnancy in 21 patients (21% incidence). The disease occurred during the first pregnancy in 38% and occurred mainly during postpartum period (79%). The unusual feature of this syndrome was the disproportionate severe renal involvement compared to the thrombocytopenia; 81% of patients required hemodialysis during the acute phase while 61% eventually reached end-stage renal disease in less than a month from the beginning of the episode. On the other hand 40% of patients had a platelet count of >100,000/mm^3^.

A variety of the therapeutic interventions have been tried with pregnancy-induced microangiopathy (and other forms of TTP) with limited success. Therapies such as steroids, antiplatelet agents, IVIg, and even splenectomy have been tried [59]. Not until plasma infusion and plasma exchange were introduced that the survival rate increased from 10% up to 80% currently [59, 64]. Plasma exchange and/or plasma infusion need to be initiated whenever there is a good suspicion of microangiopathy. In general this therapy will have to be continued to the end of delivery or throughout the postpartum period [56, 59, 61, 64]. 

## 7. MAHA Secondary to Vasculitis

MAHA has also been reported in systemic vasculitis due to a variety of causes. One of the clinical dilemmas when it comes to systemic vasculitis is the overlap of manifestations of both disorders. One of the earliest reports has been by Ross et al. [65] in 1996 when they reported the incidence of 2 cases of MAHA that was associated with a case of Wagner's granulomatosis with positive antineutrophil antibodies (ANCAs) and another case of Guillain-Barre syndrome with positive nerve biopsy for ANCA with specificity to myeloperoxidase. A more common association of systemic autoimmune disorders and MAHA is usually with systemic lupus erythematosus (SLE), with large number of case reports and small case series [62, 66–68]. 

In a single-institution large series of patients with SLE and MAHA, Kwok et al. [69] retrospectively reviewed the data on 1203 patients with SLE over a 6-year period. Twenty-six patients were found to have MAHA in addition to their SLE diagnosis-87 patients without MAHA matched for age and sex were also included as controls. The study showed that SLE is an independent risk factor for the development of microangiopathy and that this risk was associated with higher SLE disease activity index (SLEDAI). The study also showed that infection was the only independent risk factor for mortality for those TTP patients. MAHA has been also reported in a large variety of connective tissue disorders other than SLE, including systemic sclerosis, rheumatoid arthritis, dermatomyositis, and Still's disease [70, 71]. 

Similar to other conditions with microangiopathy and MAHA, ADAMTS 13 enzyme has been linked to the pathophysiology of MAHA in autoimmune disorders. Severe reduction in the enzyme activity as well as the presence of inhibitors has been consistently found in patients with SLE and other similar disorders. Since ADAMTS 13 deficiency is considered very rare in SLE and similar disorders with MAHA, testing for this enzyme is considered a good method to distinguish between the two [65, 67, 70].

## 8. Summary

MAHA is a rare disorder that has the hallmark of fragmented red blood cells and thrombocytopenia that is nonimmune in nature. MAHA is not a single disease or even a single group of disorders; rather it is a spectrum of disorders that usually present as an HUS/TTP picture. TTP/HUS is caused by a vast array of different agents (drugs, toxins, infections, pregnancy, and autoimmunity) that damage the endothelium via multiple and varied mechanisms. Functionally altered endothelium provokes intravascular platelet aggregation, resulting in the variable clinical manifestations of the syndrome. Because of the rarity of these disorders and the nonspecific clinical and laboratory features, this diagnosis (and its underlying causes) can be easily overlooked or missed leading to the high morbidity and mortality that can be seen with this problem. Therefore, when a patient presents with unexplained thrombocytopenia and a Coombs-test-negative hemolytic anemia, a presumptive diagnosis of MAHA should be considered. One of the newer advancements in the management of this disorder is the discovery of association of the ADAMTS13 enzyme's level and activity with TTP that has led to earlier and improved detection rate which was translated to better survival. No specific therapy can be claimed to cure this group of disorders, however, few interventions have been proven to be helpful (and even life saving) in some disorders such as the use of plasma infusion and plasma exchange in the management of TTP.
